# Ferritinophagy-Mediated Ferroptosis and Activation of Keap1/Nrf2/HO-1 Pathway Were Conducive to EMT Inhibition of Gastric Cancer Cells in Action of 2,2′-Di-pyridineketone Hydrazone Dithiocarbamate Butyric Acid Ester

**DOI:** 10.1155/2022/3920664

**Published:** 2022-02-21

**Authors:** Deng Guan, Wei Zhou, Huiping Wei, Ting Wang, Kangwei Zheng, Chunjie Yang, Rui Feng, Ruifang Xu, Yun Fu, Cuiping Li, Yongli Li, Changzheng Li

**Affiliations:** ^1^College of Pharmacy, Sanquan College of Xinxiang Medical University, Xinxiang, Henan, China; ^2^College of Basic Medical Science, Sanquan College of Xinxiang Medical University, Xinxiang, Henan, China; ^3^College of Basic Medical Science, Xinxiang Medical University, Xinxiang, Henan, China 453003; ^4^Experimental Teaching Center of Biology and Basic Medical Sciences, Sanquan College of Xinxiang Medical University, Xinxiang, Henan, China

## Abstract

In metastasis of cancer cells, the epithelial-mesenchymal transition (EMT) is prerequired. Ferroptosis is an iron-mediated cellular death process, but whether it involves EMT regulation remains elusive. In addition, how stress responders (Nrf2) respond to the redox alteration and cross-talking between them needs to be determined. Our data revealed that DpdtbA (2,2′-di-pyridineketone hydrazone dithiocarbamate butyric acid ester) resisted TGF-*β*1-induced EMT in gastric cancer lines (SGC-7901 and MGC-823) through ferritinophagy-mediated ROS production. Furthermore, the depletion of Gpx4 and xCT as well as enhanced lipid peroxidation indicated that DpdtbA acted as Erastin did in ferroptosis induction, which thus provided chance to explore the causal relationship between ferroptosis and EMT. Our data illustrated that ferritinophagy-mediated ferroptosis promoted the EMT inhibition. In addition, activated Nrf2 involved the regulation on both ferroptosis and EMT in response to the alteration in the cellular redox environment. In brief, ferritinophagy-mediated ferroptosis and activation of the Keap1/Nrf2/HO-1 pathway were conducive to the EMT inhibition.

## 1. Introduction

The uncontrollable propagation and migration to the nearby organs are one of the features of cancerous cells [1], while the undergoing epithelial-mesenchymal transition (EMT) is a prerequisite. During EMT, the tumor cells lose cell-cell adhesion and gain the traits of migration and invasion. At the molecular level, epithelial markers, such as ZO-1 and E-cadherin, are weakened, while vimentin and N-cadherin are increased correspondingly [2–4]. In addition, the transforming growth factor (TGF), cytokine, nuclear receptor, receptor tyrosine kinase (RTK), and reactive oxygen species (ROS) are shown to play a role in the EMT regulation [5–7]. In addition to the abovementioned, EMT in fact is in a hybrid E/M phenotype for cancer cells, and “phenotypic stability factors” (PSFs, GRHL2, OVOL2, *Δ*Np63*α*, and NUMB) can maintain the hybrid E/M phenotype [8–11], indicating that the EMT process is quite complex. Finally, EMT not only enables the multistep process leading to the colonization of distant anatomical sites but also endows malignant cells with an accrued resistance to a variety of therapeutic regimens [12].

Ferroptosis is an iron-dependent cell death, distinct from apoptosis. In ferroptosis, cell death is executed by reactive oxygen species- (ROS-) mediated peroxidation of polyunsaturated fatty acids (PUFAs), which can be reduced by glutathione peroxidase 4- (Gpx4-) mediated glutathione (GSH) [13]. Cysteine deriving from cystine reduction is used for GSH synthesis, while cystine transportation into the cell was through the system Xc-transporter SLC7A11 subunit; therefore, depletion of cysteine resulted in ferroptosis. Interestingly, Viswanathan et al. found that therapy-resistant cancer cells cross EMT and were sensitive to ferroptosis, which meant that ferroptosis inducers may be used to inhibit the EMT of cancer cells [14, 15]. In addition, NCOA4-mediated ferritin degradation in lysosomes triggered to ferroptosis induction [16], which was due to Fenton reaction-mediated ROS production. Some iron chelators were able to induce ferritinophagy in previous reports [17, 18]; however, the relationship between ferritinophagy, EMT, and ferroptosis remains unclear.

Nuclear factor erythroid 2-related factor 2 (Nrf2) is the master regulator of antioxidant and cytoprotective systems [19] and is located in the cytoplasm under normal circumstances. During oxidative stress insulting, Nrf2 is translocated to the nucleus, where it binds to the antioxidant response elements and activates its downstream target genes [20], including glutathione (GSH) synthesis, chemoresistance, and cytoprotection [21]. The homeostasis of Nrf2 is controlled by Kelch-like ECH-associated protein 1 (Keap1) to proteasomal degradation [22]. The accumulating evidence reveals that the Nrf2 plays a role in tumorigenesis [23]. In addition, reactive oxygen species (ROS) are recognized as actors in the adaptation of cancer cells to therapy, which is important in cancer cell drug resistance [22]. On the other hand, emerging evidence suggests the involvement of Nrf2 in transforming growth factor *β*1- (TGF-*β*1-) stimulated EMT in rat renal tubular cells and AECs [24]. However, the function of Nrf2 in EMT modulation remains to be determined.

Our previous work showed that “ferritinophagic flux” (NCOA4/ferritin) was a dominant driving force in determination of status of EMT in the CT26 cell line and gastric cancer cell lines in normal oxygenation [25–27]; however, cancer cells almost grow in hypoxia environment. The recent research revealed that 2,2 ′-di-pyridylketone hydrazone dithiocarbamate butyric acid (DpdtbA) also induced EMT inhibition in the hypoxia environment, which was related to activation of the p53 and PHD2/hif-1*α* pathway [28]. Since that “ferritinophagic flux” (NCOA4/ferritin) played a crucial role in determination of the status of EMT, the cellular redox response may occur. In this study, we found that DpdtbA treatment also resulted in depletion of Gpx4 and xCT and increase in lipid peroxidation, hinting there was an occurrence of ferroptosis; thus, the causal relationship between EMT and ferroptosis was explored. Our data revealed that ferroptosis induction was conducive to EMT inhibition. In addition, in response to ferritinophagy-mediated ferroptosis, the Nrf2 activation may be triggered. Interestingly, knockdown of Nrf2 by siRNA resulted in attenuation in ferroptosis induction and EMT promotion, hinting there was cross-talk between redox responder (Nrf2) and the induced cellular events. Our data strongly suggested that the ferroptosis induction and activation of the Keap1/Nrf2/HO-1 pathway were advantageous to the EMT inhibition, which highly depended on the strength of ferritinophagic flux (ROS production).

## 2. Materials and Methods

### 2.1. Materials

3-Methyladenine (3-MA), dichlorofluorescein (H_2_DCF-DA), ferrostatin-1, 4′,6-diamidino-2-phenylindole (DAPI), TGF-*β*1, N-acetyl-L-cysteine (NAC), Roswell Park Memorial Institute- (RPMI-) 1640, and other chemicals were purchased from Sigma-Aldrich (USA). Erastin was from MedChemExpress. Antibodies of vimentin, NCOA4, Nrf2, Keap1, HO-1, and gapdh for Western blotting were obtained from Proteintech Group Inc. (Wuhan, China). Antibodies of E-cadherin and ferritin (H chain) and secondary antibodies (fluorescence labeled for immunofluorescence) were purchased from Cell Signaling Technology (USA). Ferritin antibody for immunofluorescence was obtained from Santa Cruz Biotechnology (USA, Santa Cruz). NCOA4 antibody for immunofluorescence was purchased from Atlas Antibody (Sweden). Secondary antibodies for Western blotting were obtained from EarthOx, LLC (San Francisco, USA).

### 2.2. Cell Treatment

The cells (SGC-7901 and MGC-803, Yuchi Cell Biological Technology Co. Ltd.) in the exponential phase were cultured in RPMI-1640 medium supplemented with 10% fetal calf serum (FCS) and antibiotics as described previously [25]. The DpdtbA in 70% DMSO was used for cell treatment at a ratio of 1 : 100 (cell culture) in order to minimize toxicity of the solvent (DMSO final concentration ≤ 0.7%) [29]. In parallel, 70% DMSO was added to counteract the effect of the solvent when performing intergroup analysis.

### 2.3. Assay of Cellular ROS

MGC-803 (or SGC-7901) cells were treated by DpdtbA (5.0 *μ*M) or inhibitor (at indicated concentration) for 24 h. After trypsin digestion, the cells were collected by centrifugation, then resuspended in H_2_DCF-DA containing serum-free culture medium for 30 min. A flow cytometer (Becton-Dickinson, USA) was used to perform the intracellular ROS assay [25].

### 2.4. Knockdown of NCOA4 and Nrf2

Genetic knockdown of NCOA4 (or Nrf2) was performed based on the procedure as described previously [25]. The small-interfering RNA (si-RNA-mate (siN0000001-1-5) and siRNA (siG000008031A-1-5 and siG000008031B-1-5 for NCOA4; siB160506092821-1-5 and siB160729033042-1-5 for Nrf2)) were obtained from Ribobio, China. Briefly, the MGC-803 cells (1 × 10^6^) were transfected with 100 pmol of siRNA using Lipofectamine™ Stem Transfection Reagent (Invitrogen, USA) for 12 h. For immunofluorescence, confocal analysis of the MGC-803 cells was performed as described above except culturing the cells firstly in 24-well plates with cover glass.

### 2.5. Immunofluorescence Analysis

An immunofluorescence confocal analysis was conducted to investigate the changes of either EMT-related or ferritinophagy-related proteins [25]. Briefly, the MGC-803 (SGC-7901) cells on the cover glass were treated in the following steps: fixation with 4% paraformaldehyde, permeation in 0.5% Triton X-100 (30 min), and blockage in 1% BSA (1 h). Next, the cells were treated with investigated primary antibody (ferritin and H chain (Santa Cruz Biotechnology), NCOA4 (Atlas Antibodies), and vimentin and E-cadherin (Cell Signaling Technology)) at 4°C for overnight. Then, the fluorescence-labeled secondary antibody was used to associate with primary antibody. Finally, the cells were counterstained further with DAPI. The representative cells were recorded on a confocal laser scanning microscope (Nikon Eclipse Ts2, Japan) at a magnification of 400x.

### 2.6. Western Blotting Analysis

The procedure for Western blotting was as described previously [25]. The cells (MGC-803 or SGC-7901) treated by indicated agents were scraped in 200 *μ*l lysis buffer and lysed on ice for 30 min. The supernatant was collected by centrifugation. The protein concentration of the supernatant was determined by using enhanced BCA protein assay kit (Beyotime, China). Equal amounts of denatured proteins were loaded on 13~15% sodium dodecyl sulfate-polyacrylamide gels for electrophorese analysis. Next, the separated proteins on the gel were transferred to a polyvinylidene difluoride membrane (Millipore, Billerica, MA). The membrane was incubated with an appropriate primary antibody after blockage by nonfat milk at 4°C for overnight; then, the secondary antibody was added to associate the primary antibody. The protein band was visualized by using a super-sensitive ECL solution (Boster Biological Technology Co. Ltd.) on a Syngene G: BOX imager (Cambridge, United Kingdom). Quantification analysis of fluorescence intensity of the protein band was performed using ImageJ software.

### 2.7. Statistical Analysis

Results are presented as the mean ± SEM. Comparisons between multiple groups were performed by one-way ANOVA with Dunnett's post hoc correction. A *p* value < 0.05 was considered statistically significant.

## 3. Results

### 3.1. DpdtbA Treatment Resulting in Alteration in EMT-Related Proteins in Gastric Cancer Cell Line-Involved ROS Production

**Figure 1:**
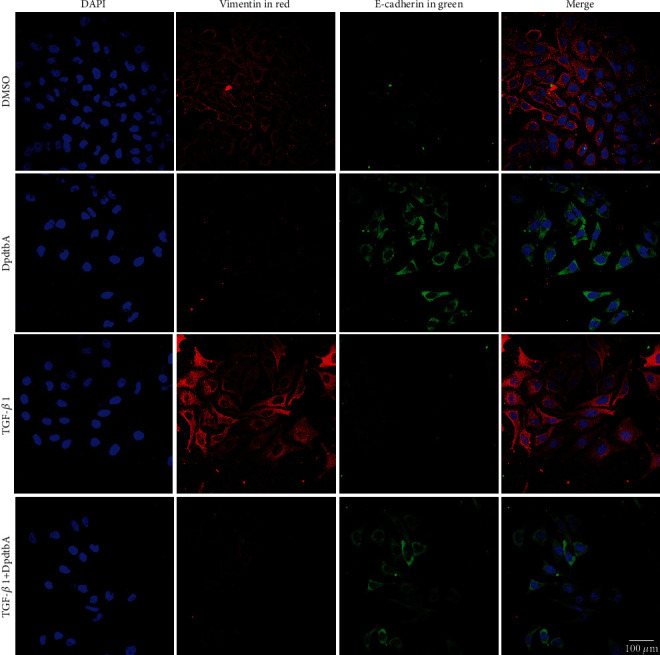
DpdtbA treatment resulted in alteration in level of EMT-related proteins in MGC-803 cell line. Objective size: 40 × 10; scale bar: 100 *μ*m.

DpdtbA (2,2′-di-pyridineketone hydrazone dithiocarbamate butyric acid ester) exhibited significant antitumor activity [30, 31]. In addition, DpdtbA treatment led to suppression of EMT-related markers under normoxia and hypoxia conditions [28]; however, the detail of inhibition of mechanism was not fully determined. To this end, a model of EMT was first established through TGF-*β*1 induction. As shown in Figure 1, TGF-*β*1 treatment resulted in the cells in stretched and fibroblast-like shape, supporting the cells undergoing EMT [30], but the addition of DpdtbA led to retraction of the cells in shape. Importantly, the EMT-related proteins were faded in red fluorescence (vimentin) and enhanced in green (E-cadherin) even in the presence of TGF-*β*1. Similar observation was in the SGC-7901 cell line (Fig. S1). Those indicated that DpdtbA was able to inhibit EMT transformation in gastric cancer lines.

ROS production was observed in EMT induction [31], but whether the EMT inhibition is also associated with the ROS production has not been fully determined. For this purpose, the alteration of cellular redox status was further determined. Thus, a ROS scavenger, N-acetyl-L-cysteine (NAC), was used to determine whether there was ROS involvement in the action of mechanism of DpdtbA. Fig. S2 shows that addition of NAC almost neutralized the effect of DpdtbA on the regulation of EMT-related proteins, hinting that the EMT inhibition was involved in ROS production.

### 3.2. Ferritinophagy-Mediated ROS Production Played a Role in DpdtbA-Induced EMT Inhibition

**Figure 2:**
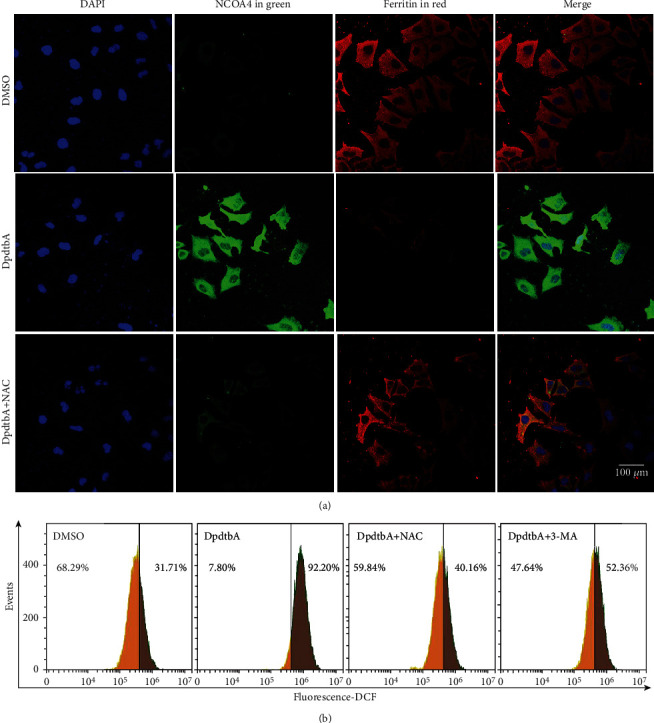
DpdtbA-induced ferritinophagy correlated with the ROS production. (a) DpdtbA-induced ferritinophagy was redox active; objective size: 40 × 10; scale bar: 100 *μ*m. (b) DpdtbA-induced ROS production involved autophagy.

Our previous study revealed that ferritinophagy was an important contributor in ROS production [18]. DpdtbA may have similar action in ferritinophagy induction.  Figure 2 shows that DpdtbA treatment caused an enhancement in green fluorescence of NCOA4, fading in red fluorescence of ferritin compared to DMSO in MGC-803 cells, indicating that a ferritinophagy occurred. Furthermore, although ferritinophagy is related to ROS production, it is not necessary in fact. DFO is a ferritinophagy inducer, but it is used as a ROS scavenger. Therefore, the redox characteristic of the induced ferritinophagy needed to be determined. For this purpose, the ROS assay was conducted via flow cytometry after the cells were stained by H_2_DCF-DA. As shown in Figure 2(b), DpdtbA treatment resulted in significant increase of ROS compared to DMSO, but addition of NAC abated markedly the inductive effect of DpdtbA on ROS induction. Similarly, addition of 3-methyladenine (3-MA), an autophagy inhibitor, also weakened the ROS production, indicating that DpdtbA-induced ROS production correlated with the occurrence of ferritinophagy. Similarly, this scene also occurred in SGC-7901 cells (Fig. S3).

**Figure 3:**
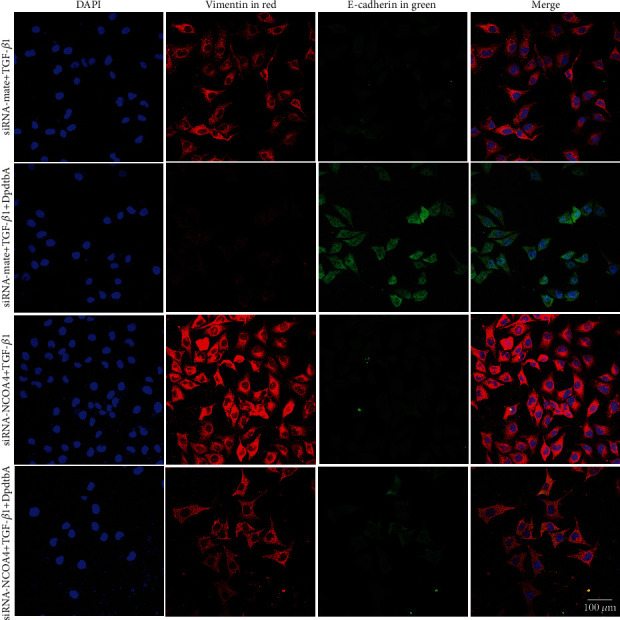
TGF-*β*1-induced EMT involved NCOA4 regulation in MGC-803 cell. Objective size: 40 × 10; scale bar: 100 *μ*m.

To further determine the contribution of ferritinophagy to the EMT inhibition, the genetic knockdown of NCOA4 was performed. As shown in Figure 3, the intensity of red fluorescence of vimentin was decreased, while the intensity of green fluorescence of E-cadherin was accordingly increased once there was DpdtbA treatment in the presence of TGF-*β*1. Interestingly, knockdown of NCOA4 gave rise to slight enhancement of vimentin in red fluorescence, prompting that NCOA4 may involve the regulation of EMT-related proteins. As a result, knockdown of NCOA4 would significantly attenuate the regulatory effect of DpdtbA on EMT-related proteins. Those indicated that NCOA4-mediated ferritinophagy contributed to the EMT inhibition, which was in accordance with our previous observations [30].

### 3.3. DpdtbA-Induced EMT Inhibition Involved Ferritinophagy-Mediated Ferroptosis

**Figure 4:**
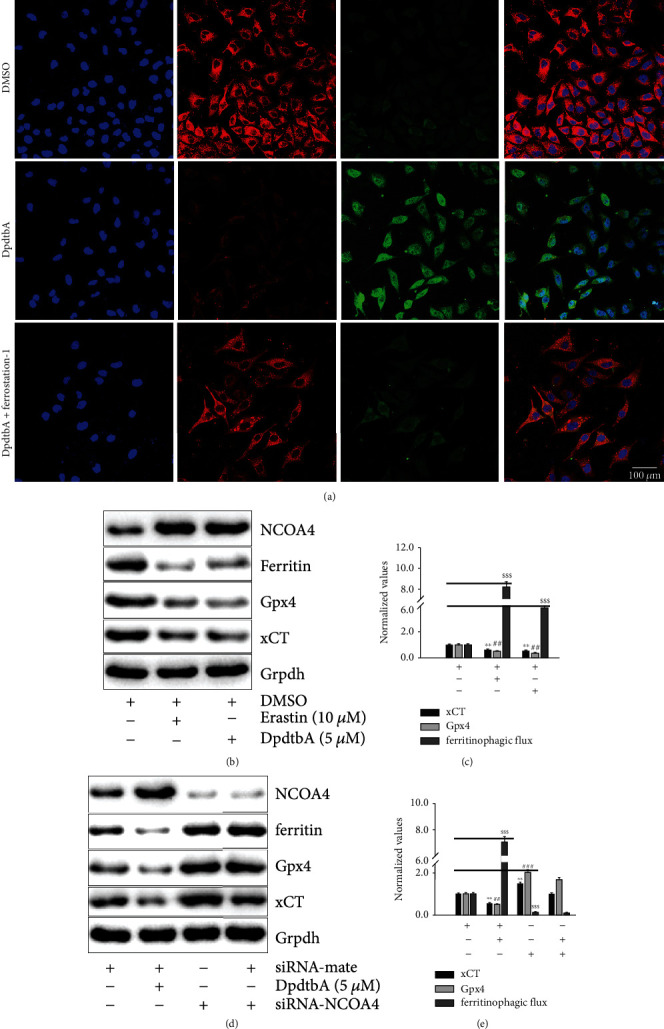
DpdtbA-induced EMT inhibition involved ferroptosis. (a) Ferrostatin-1 could attenuate the regulatory effect of DpdtbA on EMT-related proteins in MGC-803. The concentrations of Erastin and DpdtbA were as indicated, all the same, unless otherwise specified. Objective size: 40 × 10; scale bar: 100 *μ*m. (b) Both DpdtbA and Erastin treatment resulted in ferritinophagy (NCOA4-mediated ferritin degradation) and depletion of Gpx4 and xCT; (c) quantitative analysis derived from (b) indicated that the ferroptosis induction of those agents correlated with the enhanced ferritinophagic flux; (d) knockdown of NCOA4 weakened the regulatory effect of DpdtbA on ferroptosis-related proteins in MGC-803 cell; (e) quantitative analysis derived from (c). ^∗∗^^,##^*p* < 0.05 vs. control, ^∗∗∗^^,###^*p* < 0.01 vs. control, one-way ANOVA with Dunnett's post hoc correction.

Ferroptosis induction involved ROS production, while ferritinophagy-mediated ROS production contributed to the EMT inhibition, which promoted us to consider whether ferroptosis was involved in the EMT process. Thus, a ferroptosis-specific inhibitor, ferrostatin-1 (fer-1), was used to determine if this was true. As expected, the ferroptosis inhibitor indeed neutralized the regulatory effect of DpdtbA on vimentin and E-cadherin (Figure 4(a)), implying that the EMT inhibition may involve ferroptosis induction. In addition, the depletions of either selenoenzyme glutathione peroxidase 4 (Gpx4) or solute carrier family 7 member 11 (xCT) may trigger ferroptosis [32, 33]; then, their abundances were investigated. Fig. S4A shows that DpdtbA treatment caused downregulation of both Gpx4 and xCT, but addition of ferrostatin-1 markedly stopped them from decrease induced by the agent. This situation was also observed in the SGC-7901 cell (Fig. S4C). In addition to the abovementioned, lipid peroxidation and depletion of GSH were critical characteristics for ferroptosis induction [34, 35]; DpdtbA could achieve similar consequence as Erastin, a ferroptosis inducer did; and such action could be abated by the addition of ferrostatin-1 (or NAC) in Fig. S5; this further supported that the agent was able to induce ferroptosis.

As mentioned above, DpdtbA induced ferroptosis as Erastin did; the causal relation between ferritinophagy and ferroptosis needs to be determined. As shown in Figure 4(b), DpdtbA and Erastin treatment produced depletion of ferritin and upregulation of NCOA4 along with depletions of Dpx4 and xCT, clearly indicating that ferritinophagy caused ferroptosis. To reinforce the role of ferritinophagy, we used the term of ferritinophagic flux (NCOA4/ferritin).  Figure 4(c) shows that the depletions of Gpx4 and xCT correlated with enhanced ferritinophagic flux, implying that ferritinophagic flux determined the fate of ferroptosis. To corroborate the above speculation, the effect of NCOA4 on ferroptosis induction was conducted.  Figure 4(d) reveals that knockdown of NCOA4 led to upregulations of xCT, Gpx4, and ferritin; therefore, it can be imaged that such an action would chip away the regulative effect of DpdtbA on ferroptosis-related proteins. Surely either concentration of DpdtbA or efficiency of NCOA4 knockdown determined the abundance of those proteins. The quantitative analysis of the related proteins is presented in Figure 4(e) after normalization. Clearly, the data further supported the above conclusion that ferroptosis induction depended on the strength of the ferritinophagic flux.

### 3.4. DpdtbA-Induced EMT Involved Nrf2 Activation

**Figure 5:**
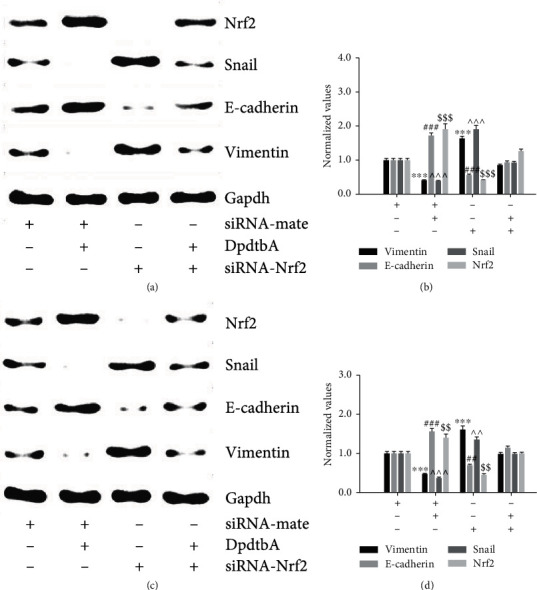
Nrf2 had a role in DpdtbA-induced EMT inhibition. (a) The EMT inhibition induced by DpdtbA was related to activation of Nrf2 in MGC-803 cells. (b) Quantitative analysis derived from (a) after normalization. (c) DpdtbA-induced EMT inhibition was achieved through activation of Nrf2 in SGC-7901 cells. (d) Quantitative analysis derived from (c) after normalization. ^∗∗^^,##^*p* < 0.05; ^∗∗∗^^,###,$$$^*p* < 0.01 vs. control, one-way ANOVA with Dunnett's post hoc correction.

Since DpdtbA-induced EMT inhibition was achieved through ferritinophagy-mediated ferroptosis induction, the cellular redox environment would be changed accordingly. Nrf2, which is one of the master transcription factors, may respond to the redox change. Fig. S6 shows that the Nrf2 was upregulated when the cell was exposed to DpdtbA, implying that Nrf2 might involve the EMT regulation. To this end, the Nrf2 was knocked down to assay its effect on EMT regulation. As shown in Figure 5(a), knockdown of Nrf2 led to obvious increases of abundance of snail and vimentin, while DpdtbA treatment caused those proteins to be downregulated; therefore, a rescued result would be expected in the combination treatment of Nrf2 knockdown with DpdtbA. Those data clearly supported that Nrf2 also involved EMT regulation in the MGC-803 cell. The quantitative analysis derived from Figure 5(a) is presented in Figure 5(b) after normalization. Clearly, the changes for the indicated proteins in Figure 5(b) were significant in individual treatment, not in combination treatment. A similar situation occurred in the SGC-7901 cell (Figures 5(c) and 5(d)). Those suggested that the activation of Nrf2 was conducive to the inhibition of EMT, inconsistent with what was reported previously [36].

### 3.5. Nrf2 Activation Was Related to Ferroptosis Induction

**Figure 6:**
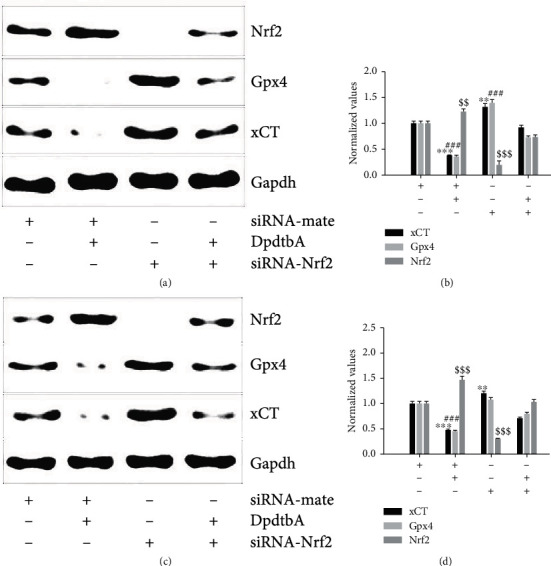
DpdtbA-induced ferroptosis involved Nrf2 activation. (a) The downregulation of Gpx4 and xCT correlated with activation of Nrf2 in MGC-803 cells; (b) quantitative analysis derived from (a) after normalization; (c) the downregulation of Gpx4 and xCT correlated with activation of Nrf2 in SGC-7901 cells; (d) quantitative analysis derived from (c) after normalization. ^∗∗^^,##^*p* < 0.05; ^∗∗∗^^,###,$$$^*p* < 0.01 vs. control, one-way ANOVA with Dunnett's post hoc correction.

It has been demonstrated that activation of Nrf2 induces the expression of multiple defensive genes in transcription, including xCT that serves to cope with ferroptosis [34]. Similarly, Gpx4 is responsible for reduction of peroxidation of polyunsaturated fatty acids, and its expression was regulated by chaperone-mediated autophagy [37]. The depletion of either xCT or Gpx4 triggers to ferroptosis. Thus, the correlation of activation of Nrf2 with Gpx4 and xCT was further investigated.  Figure 6 shows that DpdtbA treatment resulted in depletions of Gpx4 and xCT and activation of Nrf2; nevertheless, Nrf2 knockdown significantly attenuated the regulatory effect of DpdtbA on ferroptosis-related proteins in MGC-803 cells, indicating that Nrf2 also involved the regulation of ferroptosis (Figure 6(a)). The comparative analysis was performed after normalization (Figure 6(b)). Similar observation occurred in SGC-7901 cells (Figures 6(c) and 6(d)). Those all indicated that Nrf2 as a redox-sensitive transcriptional factor was implicated in ferroptosis induction. Next, the degradation manner of Gpx4 and xCT was further investigated because either ubiquitin-proteasome system (UPS) or autophagy controls their stability. Fig. S6 shows that the addition of 3-MA could attenuate the regulatory effect of DpdtbA on Gpx4 and xCT, implying that DpdtbA-induced autophagy was in charge of the dynamic equilibrium of Gpx4 and xCT.

### 3.6. Ferritinophagy-Mediated ROS Production Was Responsible for Activation of Keap1/Nrf2/HO-1 Pathway

**Figure 7:**
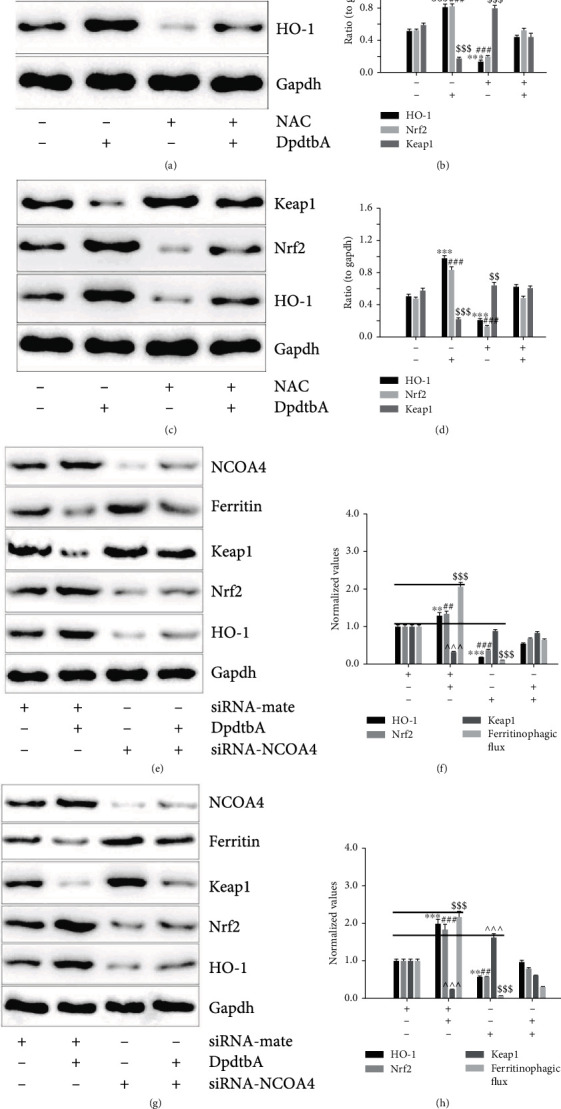
Ferritinophagy-mediated ROS production triggered the activation of Keap1/Nrf2/HO-1 pathway. (a) Keap1 downregulation resulted in activation of Nrf2 and its downstream target gene in MGC-803 cells; (b) quantitative analysis derived from (a) after normalization; (c) Keap1 downregulation resulted in activation of Nrf2 and its downstream target gene in SGC-7901 cells; (d) quantitative analysis derived from (c) after normalization; (e) the enhanced ferritinophagic flux correlated with activation of Keap1/Nrf2/HO-1 pathway in MGC-803; (f) quantitative analysis derived from (e); (g) the enhanced ferritinophagic flux correlated with activation of Keap1/Nrf2/HO-1 pathway in SGC-7901; (h) quantitative analysis derived from (g). ^∗∗^^,$$,##^*p* < 0.05; ^∗∗∗^^,###,$$$^*p* < 0.01 vs. control, one-way ANOVA with Dunnett's post hoc correction.

Since Nrf2 activation involved the EMT inhibition and ferroptosis induction, the underlying mechanism of Nrf2 activation needed to be determined. It has been demonstrated that the homeostasis of Nrf2 is controlled by Keap1 through proteasomal degradation [22]. For this reason, the level of Keap1 was determined by Western blotting.  Figure 7(a) shows that Keap1 was significantly downregulated upon DpdtbA treatment, implying that the activation of Nrf2 was due to degradation of Keap1. Next, we determined the manner of Keap1 degradation. Fig. S7 shows that addition of 3-MA or ferrostatin-1 could neutralize the regulatory effect of DpdtbA on Keap1, supporting that autophagy controlled the homeostasis of Keap1. HO-1 is a downstream gene of Nrf2, and the Nrf2 activation was accompanied by upregulation of HO-1 (Figure 7(a)). A quantitative comparison is shown in Figure 7(b). In addition, addition of NAC could attenuate the regulatory effect of DpdtbA on Keap1/Nrf2/HO-1, indicating that the cellular redox alteration caused the activation of the pathway (Figure 7(a)). Similar results were observed in SGC-7901 cells (Figures 7(c) and 7(d)). To illustrate that the ferritinophagy induction was responsible for the activation of Keap1/Nrf2/HO-1, the effect of the NCOA4 knockdown on the pathway was further investigated. As shown in Figures 7(e) and 7(g), knockdown of NCOA4 markedly attenuated the regulatory effect of DpdtbA on the proteins in the pathway, supporting that activation of keap1/Nrf2/HO-1 highly depended on the strength of the ferritinophagic flux (Figures 7(f) and 7(h)).

## 4. Discussion

Now that epithelial-mesenchymal transition (EMT) is a necessary step for metastasis of cancer cells [38, 39], the new-type EMT inhibitor needed to be developed. Dithiocarbamate derivatives exhibited significant regulatory effect on EMT-related proteins in our previous studies [28, 31, 40], but the details in the molecular level remained unclear. During EMT transformation, upregulation of vimentin and downregulation of E-cadherin were normally observed with morphological change [41, 42]; therefore, the opposite trend achieved would be good for metastasis inhibition. DpdtbA exhibited EMT inhibition like other dithiocarbamate derivatives through influencing the expression of vimentin and E-cadherin (Figure 1). In addition, ROS production was reported in EMT development [7]; however, the role of ROS in EMT inhibition was payed little attention; we reported that 2,2 ′-di-pyridine ketone hydrazone dithiocarbamatic acid (DpdtC) inhibited EMT via through massive ROS production, i.e., a manner of “attacking poison with poison” [28]. As an analog of DpdtC, DpdtbA-inhibited EMT may be through a similar way. Thus, the redox state of the cells before and after treatment was investigated. As expected, DpdtbA treatment caused massive ROS production. Next, the origin of ROS production was further determined. As we know, mitochondria-derived ROS is in first place, but degradation of iron-containing proteins in lysosomes (or proteasomes) is also an additional ROS source. The present study revealed that DpdtbA treatment led to an occurrence of ferritinophagy, which was responsible for the ROS production (Figure 2). NCOA4 knockdown cancelled out the regulatory effect of DpdtbA on EMT, further supporting that NCOA4-mediated ferritinophagy contributed to the EMT inhibition (Figure 3). Those indicated that EMT inhibition could be achieved through generating massive ROS, similar to that reported previously [28]. This again supported the hypothesis that the strength of the ferritinophagic flux may determine the status of EMT.

Iron-mediated reactive oxygen species (ROS) production leads to lipid peroxidation and cell death in Erastin-induced ferroptosis [32, 33, 43]. To deal with the increased lipid peroxides, GSH peroxidase 4 (Gpx4) catalyzes the GSH-dependent reduction of membrane lipid peroxides [44]; thus, Gpx4 downregulation (deletion) activates the ferroptotic cascade. The depletions of Gpx4, xCT, and GSH as well as enhanced lipid peroxidation in the present study suggested that there was an occurrence of ferroptosis in the action of mechanism of DpdtbA [44]. Recently, the relationship between ferroptosis and EMT has received widespread attention; however, it is far from clear for the contrary observations [45–47]. Our results demonstrated that ferroptosis induction favored EMT inhibition in gastric cancer cells (Figure 4), or this was related to ferritinophagy-mediated ROS production. This discrepancy in the effect of ferroptosis on EMT may be related to ROS sources and the cell line specificity.

**Figure 8:**
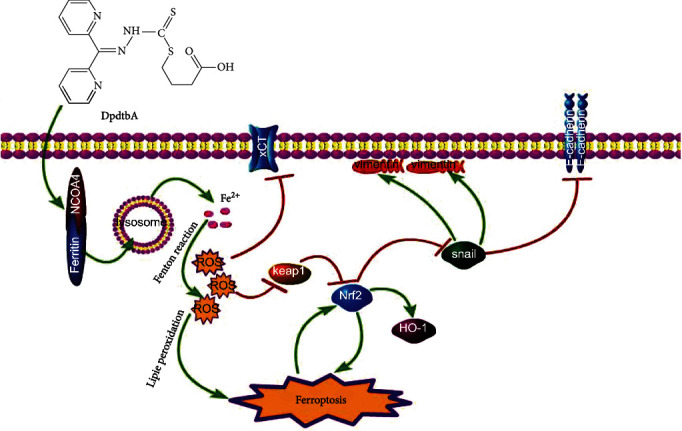
Ferritinophagy-mediated ferroptosis induction contributed to the EMT inhibition was through activation of Nrf2 in action of mechanism of DpdtbA. Ferritinophagy induction caused ROS production, accordingly triggered to ferroptosis and Nrf2 response. The activation of Nrf2 led to downregulation of snail, ultimately achieving the EMT inhibition.

Nuclear factor E2-related factor 2 (Nrf2) is the key regulatory factor required for cells to maintain an oxidative steady state [48]. DpdtbA induced EMT inhibition along with Nrf2 accumulation and depletion of snail, hinting that Nrf2 influenced the transcription factor of EMT transformation, and this concept is reinforced with knockdown of Nrf2 (Figure 5). This indicated that Nrf2 activation involved the EMT inhibition, in accordance with what were reported previously [49, 50]. However, the contrary effect of Nrf2 activation on EMT in hepatocellular carcinoma cells was observed [51]; this may reflect the yin/yang aspect of Nrf2 signaling [52]. In addition, Nrf2 also plays a role in ferroptosis regulation. Many of the key iron storage, metabolism, and transport proteins are regulated by Nrf2 [53], while the bulk of the iron-related Nrf2 target genes serve antiferroptotic functions; therefore, Nrf2 can mitigate lipid peroxidation and ferroptosis [54]. But there is some evidence in the literature of Nrf2 downstream targets that promote the ferroptotic cascade [55], including our observation (Figure 6). This double-edged (yin/yang of aspects) Nrf2 may lie in its regulation of not only iron processing but also autophagy activation proteins needed for ferritinophagy occurrence. Nrf2 plays a crucial role in mediating ferritinophagy-dependent activation of ferroptosis [54]. Cytosolic repressor protein Keap1 is known to be in charge of the level of Nrf2 through formation of the Nrf2-Keap1 complex for ubiquitination degradation of Nrf2 [56]. Nrf2 has several target genes, including intracellular redox-balancing proteins like heme oxygenase-1 (HO-1), glutathione peroxidases (GPX), and SLC7A11 [57, 58]. Heme oxygenase-1 (HO-1) induction by Nrf2 protects against the cytotoxicity of various oxidative stresses and inflammatory response, and HO-1 is regulated by the Keap1/Nrf2/HO-1 pathway [59]. The current study showed that DpdtbA-induced activation of Nrf2 was due to autophagic degradation of keap1 (Figure 7) [60, 61]. On the other hand, human Keap1 contains 27 cysteines, and the unique structure (composition) makes it more vulnerable to electrophilic modification and redox sensitive, which leads to escape of Nrf2 from their complex [62]. The ferritinophagy-mediated ROS production did not only cause ferroptosis and protein modification; therefore, activation of Nrf2 derived from Keap1 modification cannot be excluded. Nrf2 may play a crucial role in mediating ferritinophagy-dependent activation of ferroptosis [55]. The current study showed that occurrence of ferroptosis was derived from ferritinophagy induction, whereas Nrf2 activation led to upregulation of HO-1, being advantageous to ferroptosis induction [63]. Together, the action of the mechanism of DpdtbA in EMT inhibition could be proposed based on the presented data (Figure 8). Surely, Nrf2 serving either an anti- or proferroptotic role may also depend on the cell lines used. To solve the mystery on Nrf2 requires more studies.

Taken together, DpdtbA-induced EMT inhibition involved ferritinophagy-mediated ROS production and ferroptosis induction. Equally, along with those cellular events, Nrf2 was activated in response to the alteration in cellular redox environment; conversely, Keap1/Nrf2/HO-1 signaling also regulated EMT and ferroptosis. In brief, our data strongly suggested that the fate of ferroptosis, EMT status, and activation of the Keap1/Nrf2/HO-1 pathway highly depended on the strength of ferritinophagic flux (or ferritinophagy-mediated ROS production).

## Data Availability

All data are available in the manuscript.

## Abbreviations

EMT: Epithelial-mesenchymal transition
ROS: Reactive oxygen species
Nrf2: Nuclear factor erythroid 2-related factor 2
DpdtbA: 2,2 ′-Di-pyridine ketone hydrazone dithiocarbamate butyric acid
NAC: N-Acetyl-L-cysteine
NCOA4: Nuclear receptor coactivator 4.

## Supplementary Materials

Supplementary Materials Fig. S1: DpdtbA could reverse TGF-*β*1-induced EMT in SGC-7901 cell line. Objective size: 40 × 10; scale bar: 100 *μ*m. Fig. S2: NAC could significantly attenuate DpdtbA-induced EMT inhibition in MGC-803 cell. Objective size: 40 × 10; scale bar: 100 *μ*m. Fig. S3: NAC could markedly neutralize DpdtbA-induced ferritinophagy in SGC-7901 cells. Objective size: 40 × 10; scale bar: 100 *μ*m. Fig. S4: DpdtbA treatment resulted in downregulation of ferroptosis-related proteins. (A) Addition of ferrostatin-1 attenuated the regulatory effect of DpdtbA on ferroptosis-related proteins in MGC-803 cell; (B) quantitative analysis derived from (A); (C) addition of ferrostatin-1 attenuated the regulatory effect of DpdtbA on ferroptosis-related proteins in SGC-7901 cell; (D) quantitative analysis derived from (C). ^∗∗∗^^,###^*p* < 0.01 vs. control, one-way ANOVA with Dunnett's post hoc correction. Fig. S5: DpdtbA treatment resulted in depletion of GSH and enhanced lipid peroxidation. (A) Changes of GSH when the cells are exposed to the agents; (B) the abundance of lipid peroxidation in absorbance was caused by the indicated agents. ^∗∗∗^^,###^*p* < 0.01 vs. control, one-way ANOVA with Dunnett's post hoc correction. Fig. S6: DpdtbA treatment led to autophagic degradation of Gpx4 and xCT. (A) Western blot analysis; (B) quantitative analysis derived from (A). ^∗∗^^,##^*p* < 0.05 vs. control, one-way ANOVA with Dunnett's post hoc correction. Fig. S7: autophagic degradation of keap1 was responsible for activation of Nrf2. (A) Western blot analysis; (B) quantitative analysis derived from (A). ^∗∗^^,##^*p* < 0.05 vs. control, one-way ANOVA with Dunnett's post hoc correction.
