# Supplementary material for: Ferritinophagy-Mediated Ferroptosis and Activation of Keap1/Nrf2/HO-1 Pathway Were Conducive to EMT Inhibition of Gastric Cancer Cells in Action of 2,2′-Di-pyridineketone Hydrazone Dithiocarbamate Butyric Acid Ester
Source: Oxid Med Cell Longev. 2022 Feb 21;2022:3920664. doi: 10.1155/2022/3920664 (PMC8885181; doi:10.1155/2022/3920664)
Supplement: Supplementary Materials — Fig. S1: DpdtbA could reverse TGF-β1-induced EMT in SGC-7901 cell line. Objective size: 40 × 10; scale bar: 100 μm. Fig. S2: NAC could significantly attenuate DpdtbA-induced EMT inhibition in MGC-803 cell. Objective size: 40 × 10; scale bar: 100 μm. Fig. S3: NAC could markedly neutralize DpdtbA-induced ferritinophagy in SGC-7901 cells. Objective size: 40 × 10; scale bar: 100 μm. Fig. S4: DpdtbA treatment resulted in downregulation of ferroptosis-related proteins. (A) Addition of ferrostatin-1 attenuated the regulatory effect of DpdtbA on ferroptosis-related proteins in MGC-803 cell; (B) quantitative analysis derived from (A); (C) addition of ferrostatin-1 attenuated the regulatory effect of DpdtbA on ferroptosis-related proteins in SGC-7901 cell; (D) quantitative analysis derived from (C). ∗∗∗,###p < 0.01 vs. control, one-way ANOVA with Dunnett's post hoc correction. Fig. S5: DpdtbA treatment resulted in depletion of GSH and enhanced lipid peroxidation. (A) Changes of GSH when the cells are exposed to the agents; (B) the abundance of lipid peroxidation in absorbance was caused by the indicated agents. ∗∗∗,###p < 0.01 vs. control, one-way ANOVA with Dunnett's post hoc correction. Fig. S6: DpdtbA treatment led to autophagic degradation of Gpx4 and xCT. (A) Western blot analysis; (B) quantitative analysis derived from (A). ∗∗,##p < 0.05 vs. control, one-way ANOVA with Dunnett's post hoc correction. Fig. S7: autophagic degradation of keap1 was responsible for activation of Nrf2. (A) Western blot analysis; (B) quantitative analysis derived from (A). ∗∗,##p < 0.05 vs. control, one-way ANOVA with Dunnett's post hoc correction. [file 3920664.f1.docx]

**Ferritinophagy-mediated ferroptosis and activation of Keap1/Nrf2/HO-1 pathway were conducive to EMT inhibition of gastric cancer cells in action of 2,2’-di-pyridineketone hydrazone dithiocarbamate butyric acid ester**

Deng Guan^1,2,4#^, Wei Zhou^2#^, Huiping, Wei^2#^, Ting Wang^3^, Kangwei Zheng^1^, Chunjie Yang^1^, Rui Feng^3^, Ruifang Xu^3^, Yun Fu^4^, Cuiping Li^4^, Yongli Li^2*^, Changzheng Li^1,3,4*^

^1^College of Pharmacy, Sanquan College of Xinxiang Medical University, Xinxiang, Henan, P. R. China;

^2^College of Basic Medical Science, Sanquan College of Xinxiang Medical University, Xinxiang, Henan, P. R. China;

^3^Experimental Teaching Center of Biology and Basic Medical Sciences, Sanquan College of Xinxiang Medical University, Xinxiang, Henan, P. R. China;

^4^College of Basic Medical Science, Xinxiang Medical University, Xinxiang, Henan, P. R. China, 453003

**Supplementary Materials**

**DpdtbA induced EMT inhibition in SGC-7901 cells**

As shown in Fig. 1, DpdtbA exhibited EMT inhibition in MGC-803 cells. To determine whether this phenomenon is representative in gastric cancer cells, an additional cell line, SGC-7901 was used. As expected, DpdtbA could upregulate the level of E-cadherin and downregulate the level of vimentin in the presence of TGF-β1, supporting that it acted as an EMT inhibitor (Fig. S1). In addition, no obvious change in cytoskeleton of SGC-7901 cells has been noticed when TGF-β1 treatment, the stretched and fibroblast-like shape was not observed. The subtle difference in cytoskeleton between the two lines may origin from different source.


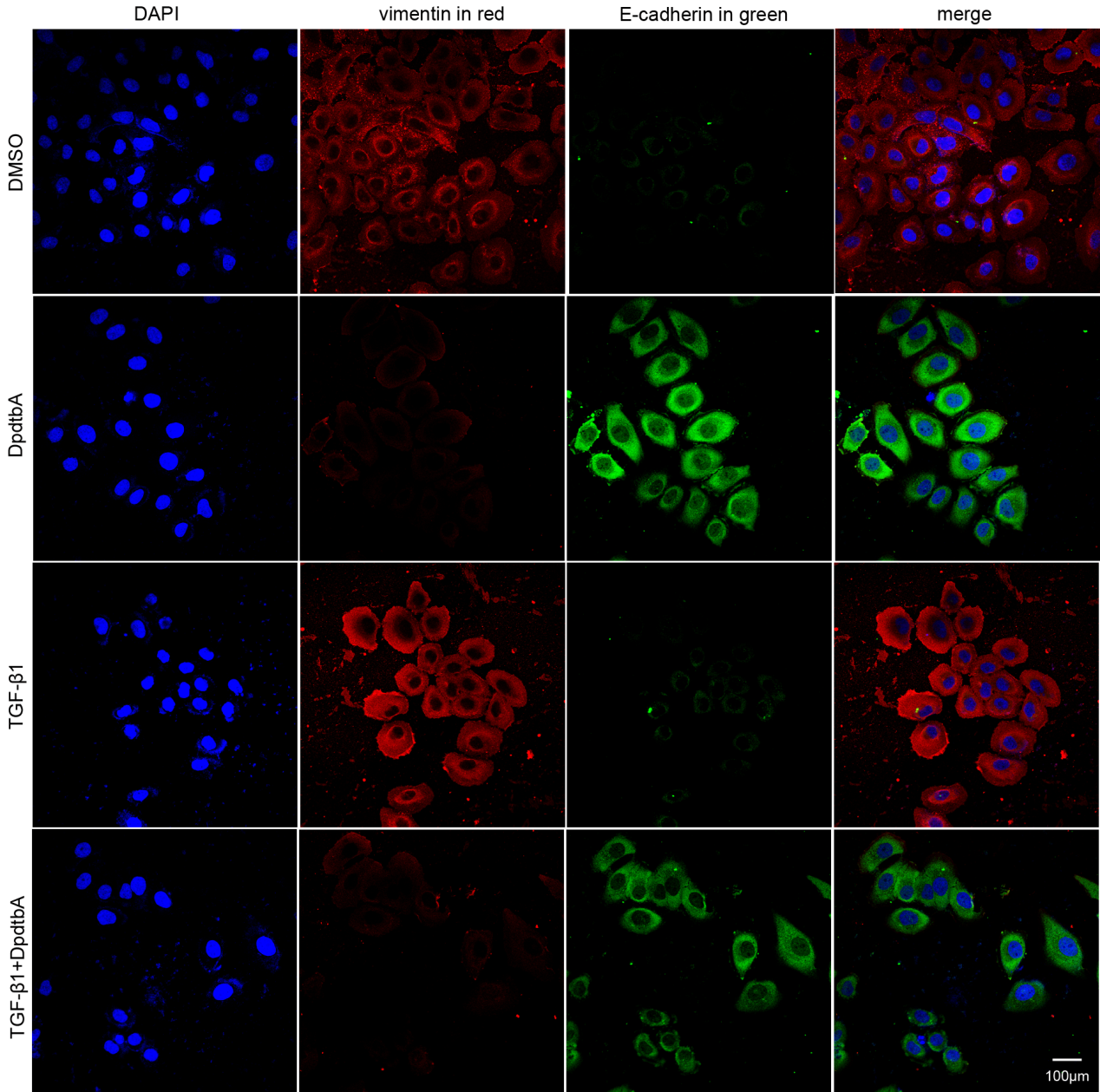


**Fig. S1** **DpdtbA could reverse TGF-β1 induced EMT in SGC-7901 cell line.** Objective size: 40 ×10; scale bar: 100 μm.

**DpdtbA induced EMT inhibition in SGC-7901 cells was related to ROS production**

It was reported that TGF-β1 induced EMT involved ROS production [1], but if EMT suppression was also similar situation? To this end, the ROS scavenger, N-acetyl-L-cysteine (NAC) was used to determine whether ROS involved. As shown in Fig. S2, addition of NAC almost neutralized the regulatory effect of DpdtbA on EMT-related markers, implying that the EMT inhibition involved ROS production (Fig. S2).


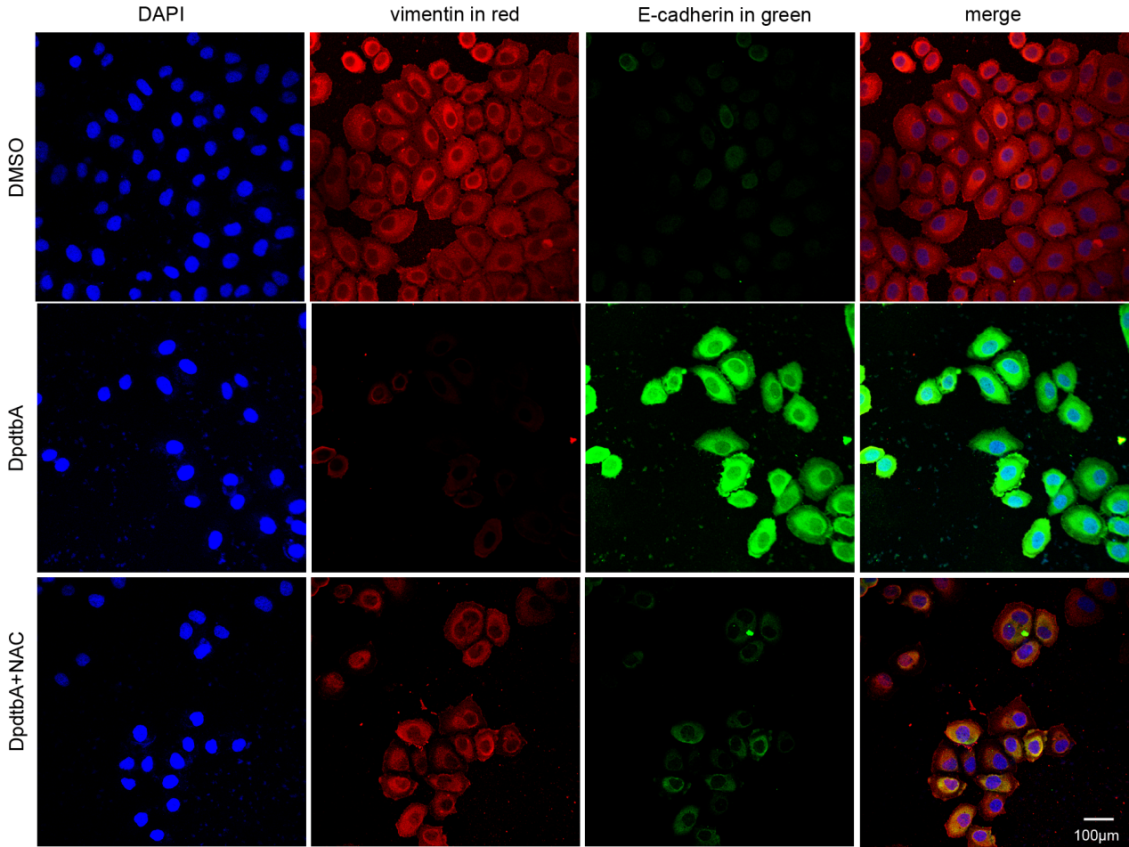


**Fig. S2 NAC could significantly attenuate DpdtbA induced EMT inhibition in MGC-803 cell.** Objective size: 40 ×10; scale bar: 100 μm.

**DpdtbA induced an occurrence of ferritinophagy in SGC-7901**

Similarly, DpdtbA treatment also led to ferritinophagy in SGC-7901 cells as it acted in MGC-803 cell line. The addition of NAC could significantly attenuate the effect of the agent on ferritinophagy induction, indicating that the ferritinophagy was redox active.


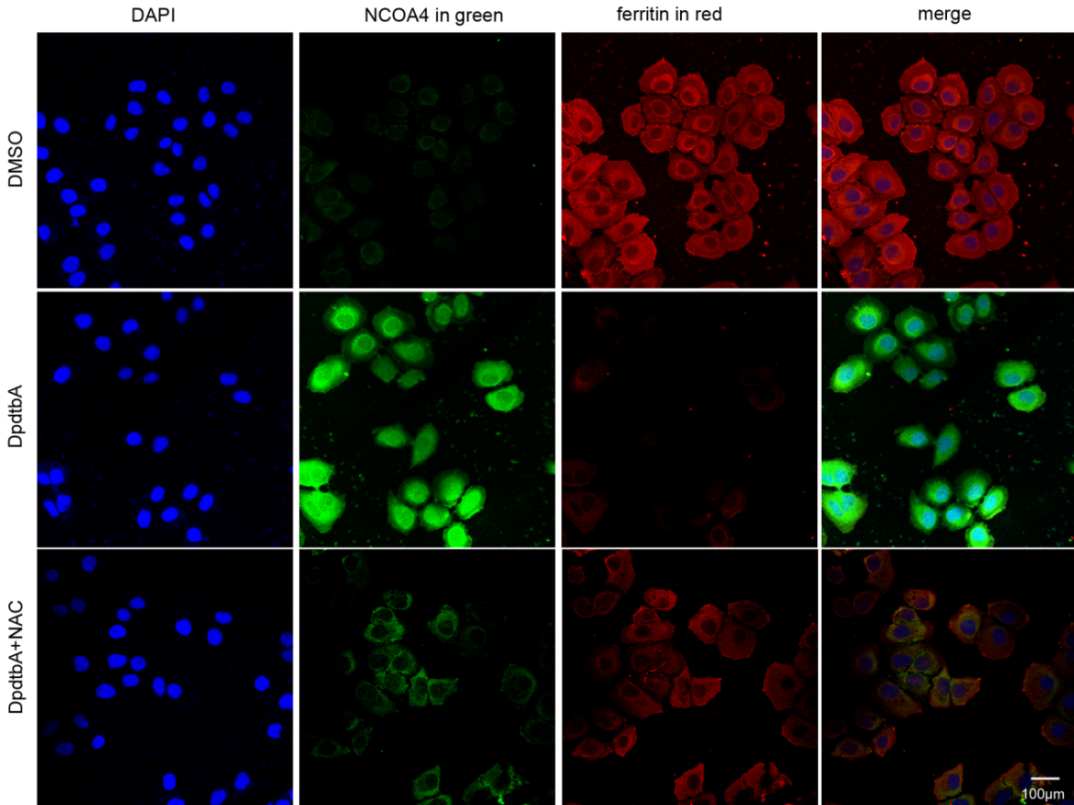


**Fig. S3 NAC could markedly neutralize DpdtbA induced ferritinophagy in SGC-7901 cells.** Objective size: 40 ×10; scale bar: 100 μm.

**DpdtbA treatment resulted in an occurrence of ferroptosis**

It was well documented that ferroptosis is a type of autophagy-dependent cell death [2], involves ferritinophagy [3]. At the heart of ferroptosis is the selenoenzyme glutathione peroxidase 4 (Gpx4) which is the most central downstream ferroptosis regulator [4]. In addition, cystine-glutamate antiporter (system xc^_^) is a heterodimeric amino acid transporter consisting of the cell surface antigen heavy chain (4F2) and the solute carrier family 7 member 11 (xCT) light chain that are linked by an intermolecular disulfide bond, controlling take-up of cystine and releases of glutamate [5]. To determine whether there was involvement of ferroptosis, the levels of Gpx4 and xCT were determined by western blotting. As shown in Fig. 4A, DpdtbA treatment caused depletion of Gpx4 and xCT, but addition of ferrostatin-1 markedly attenuated the regulatory effect of the agent on those proteins in MGC-803 cell. Similar scene occurred in SGC-7901 cell (Fig. 4C). Those suggested that DpdtbA was able to induce ferroptosis. A quantitative analysis derived from Fig. 4A (or 4C) was presented in Fig. 4B (or 4D), clearly the decrease of Gpx4 or xCT was significance in statistics (p < 0.01).

**
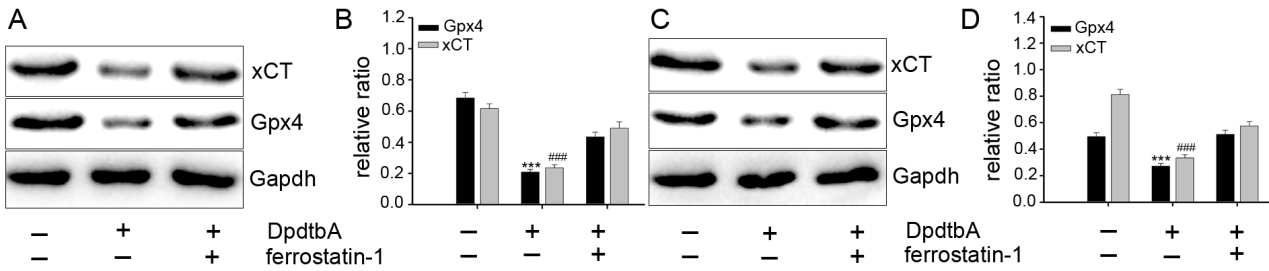
**

**Fig. S4 DpdtbA treatment resulted in downregulation of ferroptosis-related proteins.** (**A**) Addition of ferrostatin-1 attenuated the regulatory effect of DpdtbA on ferroptosis-related proteins in MGC-803 cell; （**B**）quantitative analysis derived from (**A**); (**C**) Addition of Ferrostatin-1 attenuated the regulatory effect of DpdtbA on ferroptosis-related proteins in SGC-7901 cell; （**D**）quantitative analysis derived from (**C**). ^***,###^ p < 0.01 vs control, one way ANOVA with Dunnett post-hoc correction.

**DpdtbA treatment resulted in lipid peroxidation and depletion of GSH**

A spectrophotometry was used to perform the lipid peroxidation assay, in which the ferrous ion is oxidized by lipid hydroperoxides to the ferric ion and subsequently reacts with thiocyanate to form a colored complex [6]. Briefly, the trypsinized cells were collected and treated with the DpdtbA for 24 h. Following removing the supernatant, the cells were washed with PBS. The peroxidized lipids were extracted using deoxygenated CHCl_3_/MeOH (2:1, v/v mixture; 1,000 μl) from the cells, and the lipids were transferred to a 5 ml volumetric flask, which contained 100 μl of ferrous sulfate (0.2 M HCl) and 100 μl of 3% deoxygenated thiocyanate (methanol) for 60 min. Finally, deoxygenated CHCl_3_/MeOH solvents were added to the given volume. The absorbance at 500 nm was measured using a UV-2450 spectrophotometer (Shimadzu Corporation). The molar absorptivity of the ferric thiocyanate complex expressed per mol of LOOH was determined to be 58,440 M^-1^ cm^-1^ [6]. The GSH assay was performed based on protocol described previously [7]. Briefly, the cells either treated with DpdtbA or without treatment were collected and lysed in an isosmotic solution. The lysate was further treated further and the precipitate was centrifuged. Next, 100 μl of cellular supernatant was mixed with 25 μl DTNB and 100 μl buffer, and the mixture was incubated for 5 min at room temperature. The absorbance at 405 nm that correlates GSH concentration was measured with an ELISA reader (Benchmark Plus Reader; Bio-Rad Laboratories, Inc.).

As shown in Fig. S5A, the abundance of reduced GSH was significantly decreased upon DpdtbA or Erastin treatment, in accordance with increase of ROS production. However, addition of ferrostatin-1(fer-1) markedly attenuated the regulatory effect of DpdtbA on GSH, indicating that ferroptosis was involved in the action of mechanism of DpdtbA. Since DpdtbA led to depletion of GSH, the lipid peroxidation would occur. Fig S5B showed that the level of peroxidized lipids (in absorbance) was significantly increased. However, addition of ferroptosis inhibitor, ferostatin-1 significantly decreased the level of lipids peroxidation caused by DpdtbA. In addition, addition of NAC also led to a decrease of lipid peroxidation. Those indicated that ferritinophagy-mediated ROS production resulted in the lipid peroxidation and ferroptosis induction.


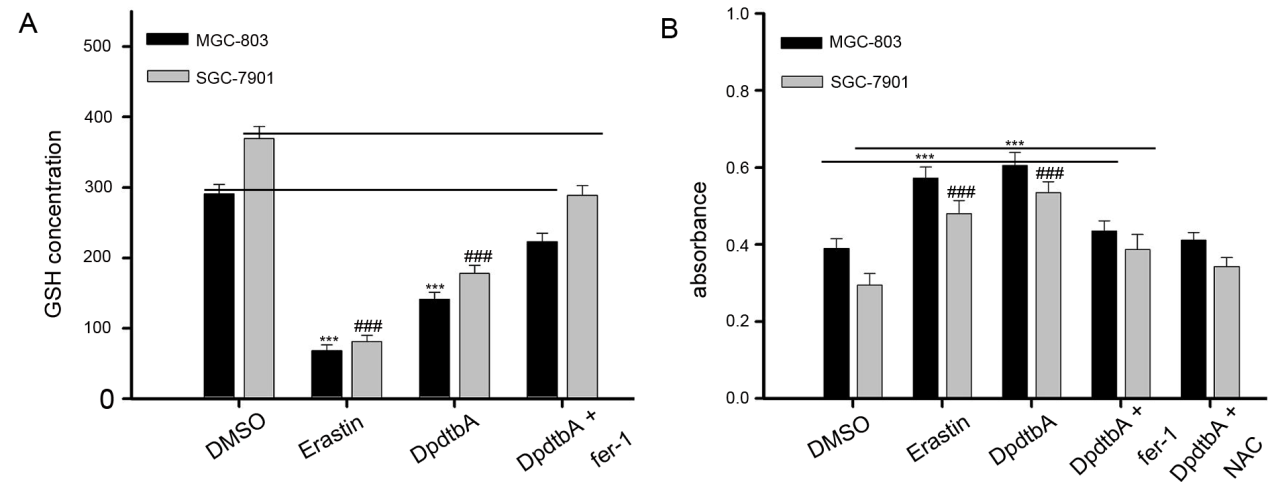


**Fig. S5 DpdtbA treatment resulted in depletion of GSH and enhanced lipid peroxidation.** (**A**) changes of GSH when the cells exposure to the agents; (**B**) the abundance of lipid peroxidation in absorbance was caused by the indicated agents. ^***,###^ p < 0.01 vs control, one way ANOVA with Dunnett post-hoc correction.

**DpdtbA treatment resulted in depletion of Gpx4 and xCT through autophagic degradation**

Ubiquitination and autophagy play crucial role to maintain homeostasis of cellular proteins. The downregulation of Gpx4 and xCT promoted us to explore underlying mechanism of depletion of them. For this reason, the 3-MA was added to assess the possible effect of it on those genes when DpdtbA treated the MGC-803 cells. Fig. S6 showed that DpdtbA treatment resulted in depletions of Gpx4 and xCT, contrary to DpdtbA, 3-MA treatment led to upregulation of them, therefore, addition of 3-MA markedly weaken the regulatory effect of DpdtbA on Gpx4 and xCT could be expected. The quantitative analysis was presented in Fig. S6B. This clearly indicated that the enhanced autophagy (ferritinophagy) was mainly in charge of the levels of both Gpx4 and xCT.


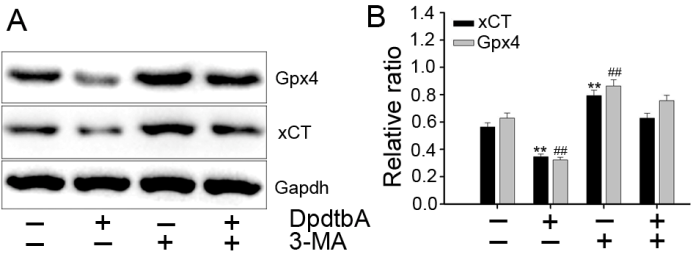


**Fig. S6 DpdtbA treatment led to autophagic degradation of Gpx4 and xCT.** (**A**) western blot analysis; (**B**) quantitative analysis derived from (**A**). ^**,##^ p < 0.05 vs control, one way ANOVA with Dunnett post-hoc correction.

**DpdtbA induced autophagy played important role in homeostasis of Keap1**

Keap1 was reported to assist in ubiquitin aggregate clearance through autophagy [8]. DpdtbA treatment resulted in downregulation of keap1, whether the degradation of keap1 was due to enhanced autophagy. For this reason, the 3-MA was added to assess whether it affected the action of DpdtbA. Fig. S7 showed that addition of 3-MA significantly attenuated the depletion of Keap1 induced by DpdtbA, indicating that homeostasis of Keap1 was partly controlled by autophagy. Furthermore, addition of ferrostatin-1 had similar consequence as addition of 3-MA, suggesting that the stability of Keap1 was partly governed by ferroptosis or ferritinophagy because the cellular redox environment influenced the redox status of Keap1. The quantitative analysis was presented in Fig. S7B.


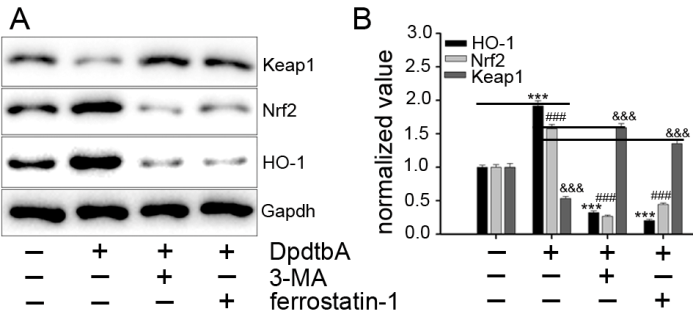


**Fig. S7 Autophagic degradation of keap1 was responsible for activation of Nrf2.** (**A**) western blot analysis; (**B**) quantitative analysis derived from (**A**). ^**,##^ p < 0.05 vs control, one way ANOVA with Dunnett post-hoc correction.

**Reference**

1. Z.J. Xu, J.K. Feng, Y.L. Li, et al., “The vicious cycle between ferritinophagy and ROS production triggered EMT inhibition of gastric cancer cells was through p53/AKT/mTor pathway”, Chemo-Biological Interactions, 2020, vol. 328, pp. 109196.
2. B. Zhou, J. Liu, R. Kang, et al., “Ferroptosis is a type of autophagy-dependent cell death”, Seminars in Cancer Biology, 2020, vol. 66, pp. 89-100.
3. M. Gao, P. Monian, Q. Pan, et al., “Ferroptosis is an autophagic cell death process”, Cell Research, 2016, vol. 26, no. 9, pp. 1021-1032.
4. W.S. Yang, R. SriRamaratnam, M.E. Welsch, et al., “Regulation of ferroptotic cancer cell death by GPX4”, Cell, 2014, vol. 156, no.1-2, pp. 317-331.
5. S. Doll and C. Marcus, “Iron and ferroptosis: A still ill-defined liaison”, IUBMB life. 2017, vol. 69, no.6, pp. 423-434.
6. A.C. Gasparovic, M. Jaganjac, B. Mihaljevic, S.B. Sunjic and N. Zarkovic, “Assays for the measurement of lipid peroxidation”, Methods Molecular Biology, 2013, vol. 965, pp. 283-296.
7. L.L. Li, H. Li, Y.L. Li, et al., “Ferritinophagy-mediated ROS production contributed to proliferation inhibition, apoptosis, and ferroptosis induction in action of mechanism of 2-pyridylhydrazone dithiocarbamate acetate”, Oxidative Medicine and Cellular Longevity, 2021, vol. 2021, pp. 5594059.
8. W. Fan, Z. Tang, D. Chen, et al., “Keap1 facilitates p62-mediated ubiquitin aggregate clearance via autophagy”, Autophagy, 2010, vol. 6, no.5, pp. 614–621.
